# Integrated machine learning and molecular dynamics framework for predicting and elucidating ABCB1 allocrite interactions

**DOI:** 10.1093/bib/bbag106

**Published:** 2026-04-07

**Authors:** Jianjia Su, Yiyang Wu, Wei Xiong, Defang Ouyang

**Affiliations:** School of Pharmacy, Shenzhen University Medical School, Shenzhen University, No. 1066 Xueyuan Avenue, Nanshan District, Shenzhen, Guangdong, 518055, China; State Key Laboratory of Mechanism and Quality of Chinese Medicine, Institute of Chinese Medical Sciences, University of Macau, Avenida da Universidade, Taipa, Macau, China; State Key Laboratory of Mechanism and Quality of Chinese Medicine, Institute of Chinese Medical Sciences, University of Macau, Avenida da Universidade, Taipa, Macau, China; School of Pharmacy, Shenzhen University Medical School, Shenzhen University, No. 1066 Xueyuan Avenue, Nanshan District, Shenzhen, Guangdong, 518055, China; State Key Laboratory of Mechanism and Quality of Chinese Medicine, Institute of Chinese Medical Sciences, University of Macau, Avenida da Universidade, Taipa, Macau, China; Faculty of Health Sciences, University of Macau, Avenida da Universidade, Taipa, Macau, China

**Keywords:** ABCB1, multidrug resistance, machine learning, molecular dynamics, allocrite interactions

## Abstract

ABCB1, a polyspecific efflux transporter, mediates multidrug resistance in cancer by interacting with diverse substrates and inhibitors, yet its recognition mechanisms remain elusive. Here, we introduce an integrated framework that synergistically combines biophysics and computational biology to predict ABCB1 allocrite interactions and elucidate their mechanisms. We curated hierarchical-confidence bioactivity datasets from multi-source assays and developed MolMM, a convolutional neural network leveraging meta-learning on noisy data and multi-task learning on refined data, achieving AUC–ROC scores of 83.33% for inhibitors and 81.26% for substrates. SHapley Additive exPlanations (SHAP) analysis revealed key molecular features, highlighting competitive polar, and hydrophobic motifs distinguishing substrates from inhibitors . Building on these ML insights, coarse-grained umbrella sampling simulations mapped these features onto free energy landscapes, proposing an amphiphilic model for substrate binding via a flip–flop process through the transmembrane pore and an inhibitory mechanism stabilizing ABCB1 in transitional conformations at the cavity’s gate. This machine learning-molecular dynamics synergy offers mechanistic insights into ABCB1 polyspecificity, facilitating rational design of inhibitors to overcome multidrug resistance.

## Introduction

 ABCB1, commonly referred to as P-glycoprotein, is an ABC transporter encoded by the *MDR1* gene. It is expressed in the plasma membrane of cells across various tissues and barriers, where it plays a key role in maintaining cellular homeostasis by effluxing xenobiotics and endogenous metabolites [1]. As a polyspecific efflux pump, it transports diverse pharmaceuticals, including anticancer drugs, antibiotics, and antivirals, thereby influencing their pharmacokinetics and bioavailability. Importantly, ABCB1 contributes to multidrug resistance (MDR) in cancer due to this polyspecificity [2]. For instance, the widespread use of ABCB1 substrate chemotherapeutic drugs, such as paclitaxel and doxorubicin, can induce the activation and overexpression of ABCB1 in cancer cells, which in turn confers resistance to these agents and contributes to treatment failure in patients. Accordingly, ABCB1 has been extensively studied in the context of MDR, including assessments to determine whether existing chemotherapeutic drugs serve as its substrates and the development of inhibitors to reverse MDR [3, 4]. However, the detailed mechanisms by which ABCB1 recognizes and binds a wide range of chemically diverse substrates and inhibitors (collectively termed allocrites) remain incompletely understood [5]. This gap hinders the rational design of targeted inhibitors and combination-based cancer therapies.

In recent years, structural and functional studies were conducted to elucidate how ABCB1 interacts with allocrites. A series of structures have revealed valuable snapshots of allocrite-bound ABCB1 conformations, offering insights into the binding of substrate drugs and small-molecule inhibitors [6–13]. These structures show that allocrites bind within the central cavity of the transmembrane domains (TMDs) in the occluded conformation, which serves as a key intermediate in the substrate translocation pathway. This allocrite binding, coupled with ATP binding and hydrolysis at the nucleotide-binding domains (NBDs), triggers essential conformational changes in ABCB1 that facilitate substrate translocation across the membrane. Furthermore, these structures demonstrate that certain inhibitors can bind in pairs, effectively arresting ABCB1 function by engaging structural features critical for its transport activity. Complementing these structural insights, functional studies—such as mutagenesis, ATPase activity assays, and efflux assays [14–17]—have investigated the roles of some specific residues in the central cavity and NBDs, which can affect substrate transport or inhibition.

However, these experimental studies are often limited by the transporter’s inherent polyspecificity and structural flexibility. For instance, conformations captured in structural studies typically represent trapped states or locked intermediates, because conformational rigidity is essential for structural determination. These strategies, which compromise the transporter’s flexibility, make it challenging to elucidate the dynamic processes of allocrite recognition and binding. Moreover, ABCB1 contains multiple overlapping binding sites within its flexible transmembrane cavity [15, 16], where perturbations at one site can trigger compensatory binding at alternative sites, thereby preserving efflux activity. This mechanism complicates the interpretation of functional assay results, as mutations or perturbations at one site may not fully abolish efflux activity, leading to ambiguous outcomes that obscure the full spectrum of allocrite interactions and hinder precise mechanistic elucidation. Computational methods have emerged as powerful complementary tools to overcome limitations of experimental approaches in ABCB1 research. From a biophysical perspective, molecular dynamics (MD) simulations provide atomistic insights into allocrite recognition and binding processes in ABCB1 [18–25]. Recently, all-atom MD simulations combined with umbrella sampling have been employed to estimate the potential of mean force (PMF) along allocrite binding pathways in ABCB1 [19, 20]. These PMF profiles provide insights into the metastable states and the microscopic transition mechanisms between them during the binding process. The PMF profiles for seven allocrites (morphine, Hoechst 33342, paclitaxel, nicardipine, rhodamine 123, tariquidar, and verapamil) reveal metastable states of allocrites binding within the transmembrane pore—formed by the encircling transmembrane helices—supporting this region as a transport-competent binding location. The free energy landscape from PMF profiles also indicates a possible binding pathway of allocrites from the aqueous phase to the binding sites through the transmembrane pore. However, PMF calculations for such complex systems demand substantial computational resources and are highly time-consuming, restricting analyses to a limited number of representative small-molecule allocrites and making it difficult to generalize to the comprehensive understanding of ABCB1’s polyspecific nature—a challenge addressed by integrating MD with computational biology techniques like machine learning (ML).

In contrast, from a computational biology perspective, ML techniques offer scalable, high-throughput predictions for potential ABCB1 substrates and inhibitors across vast chemical spaces. Ligand-based machine learning models, including neural networks, based on molecular structures or physicochemical properties have achieved high predictive accuracies, with AUC-ROC or accuracy often exceeding 80% for both substrate and inhibitor prediction (see Supplementary text subsection 1A). However, the application of current ML models remains constrained by data bottlenecks [26–28]. As a data-driven approach, ML demands large and high-quality bioactivity datasets from ABCB1 functional studies for model training and validation, which are typically assembled from multi-source in vitro assays, such as cell-based ABCB1 efflux and inhibition assays. According to the limitations in functional studies mentioned above, these assays introduce noisy labels and data heterogeneity in bioactivity datasets, undermining model generalizability and reliability (see SI text subsections 1BC).

**Figure 1:**
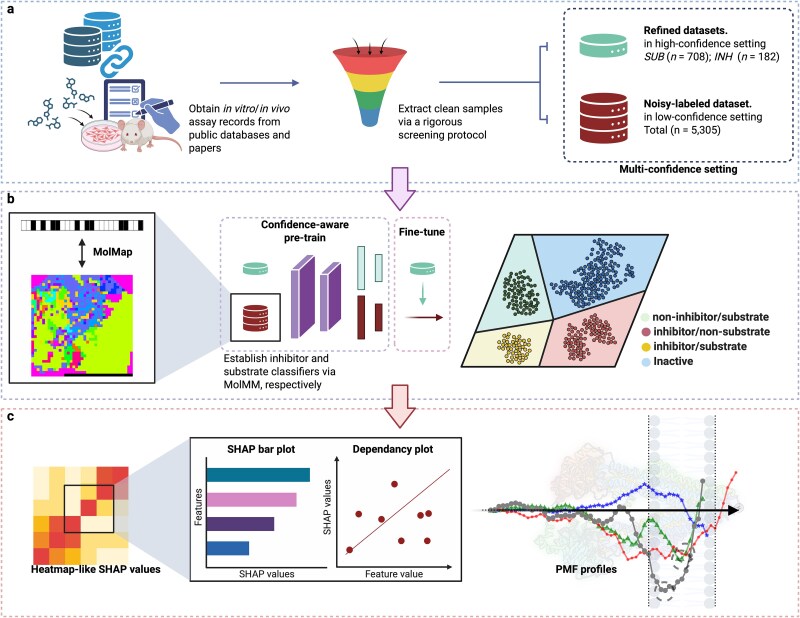
Integrated computational framework for ABCB1 substrate and inhibitor prediction. (a) Workflow for hierarchical-confidence dataset curation: systematic screening and stratification of ABCB1 substrates and inhibitors for experimental annotations from multiple assay sources. (b) Overview of the MolMM framework: integration of meta-learning and multi-task learning enables robust ABCB1 substrate and inhibitor prediction using both refined and noisy-labeled datasets. (c) Interpretation of the ABCB1-allocrite binding mechanism: combining SHAP analysis with PMF profiles from coarse-grained umbrella sampling to provide mechanistic insights into transport and inhibition.

Building on recent computational methods in ABCB1 research, we developed an integrated framework that synergistically combines biophysics and computational biology to predict ABCB1 substrates and inhibitors while elucidating their interaction mechanisms. First, to overcome the existing data bottlenecks for ML in ABCB1 bioactivity predictions, we established hierarchical-confidence bioactivity datasets including two scarce high-confidence datasets for inhibitor and substrate data, respectively (refined datasets, INH, and SUB) and a large low-confidence dataset mixing inhibitor and substrate data (noisy-labeled datasets). These datasets were generated by our hierarchical screening strategy on multiple sources of ABCB1 functional assays (Fig. 1a). Then, we proposed MolMM, a convolutional neural network (CNN)-based framework designed to handle noisy labels and data heterogeneity in our hierarchical-confidence datasets (Fig. 1b). Second, we used the SHAP method to identify discriminative molecular features that contribute to MolMM’s bioactivity prediction (Fig. 1c). Given the high dimensionality of MolMM’s input—comprising 2D Fmaps derived from 1344 molecular descriptors—we identified molecular descriptor clusters of Fmaps, and ranked the importance of them instead of individual features to capture cohesive molecular features, avoiding the loss of insights into main features amid a multitude of minor details. Moreover, we calculated the rank monotonicity between the values of individual molecular descriptors and the predicted outcomes to complement the influence directions for the molecular features to model’s prediction, enabling comprehensive understanding for allocrite recognition, and the differences between substrate and inhibitor recognition. Last, we calculated PMF profiles for four molecules with representative ABCB1 bioactivity (valproic acid, andrographolide, kaempferide, and TPGS 1000) using coarse-grained umbrella sampling simulations. These PMF profiles describe the free energy landscape of these molecules as they transition from the aqueous phase into the transmembrane pore of ABCB1 (Fig. 1c). Overall, by overlaying ML-extracted features onto these biophysical PMF landscapes, our integrated approach reveals how molecular properties drive allocrite binding dynamics, offering a unified view of ABCB1 polyspecificity that neither method achieves alone.

## Results

### Development of hierarchical-confidence ABCB1 bioactivity datasets

As a result of the screening strategy detailed in Subsection 4.1, we obtained the SUB dataset comprising 415 substrates and 293 non-substrates, along with an *in vitro* inhibitor dataset comprising 73 inhibitors and 67 non-inhibitors. Finally, by incorporating the *in vivo* inhibitor data with an identification of inhibitors (AUC$_{\mathrm{Digoxin}}$  $\ge $ 1.25-fold) and non-inhibitors (AUC$_{\mathrm{Digoxin}}$ < 1.125-fold) [29], we generated the INH dataset which included a total of 87 inhibitors and 95 non-inhibitors. Moreover, following the aggregation of refined datasets, a noisy-labeled dataset was constructed from the excluded records. The noisy-labeled dataset comprised 5305 compounds, including 3341 allocrites and 1964 non-allocrites.

**Figure 2:**
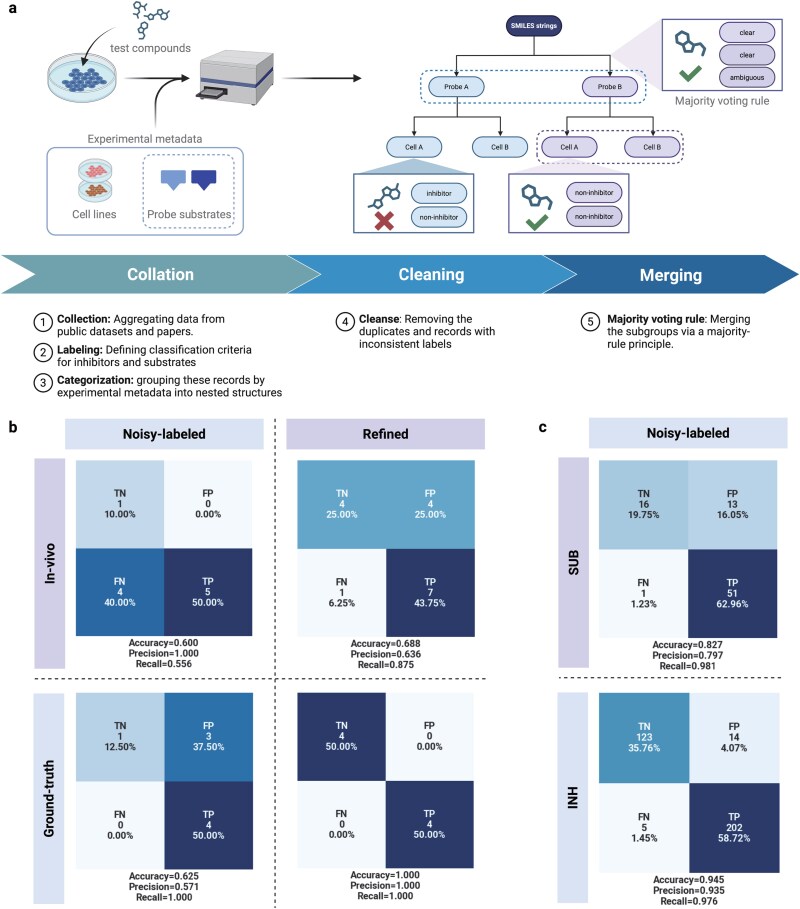
Development of hierarchical-confidence ABCB1 bioactivity datasets. (a) Overview of the data screening workflow used to construct hierarchical-confidence ABCB1 bioactivity datasets from multiple ABCB1 assay sources. (b and c) Evaluation of datasets using confusion matrices for the overlapping data between datasets. (b) Confusion matrix for inhibitor labels, illustrating the superior accuracy and precision scores of the *in vitro* INH compared with the noisy-labeled dataset, as evaluated using *in vivo* and ground-truth data. (c) Comparative matrix for refined datasets and noisy-labeled datasets for inhibitor and substrate labels, respectively.

We evaluated both the *in vitro* refined datasets and the noisy-labeled dataset using a set of compounds with detailed ABCB1 interaction annotations, referred to as the ground-truth data (GTD) (see Table 1 in ref. [20] and Table S3). For the inhibitor data, Fig. 2b indicates that the refined datasets achieved superior performance, with 100% accuracy for the overlapping eight samples (*in vitro* INH vs. GTD), compared with 62.5% accuracy and a low precision score of 57.1% for the overlapping eight samples (noisy-labeled dataset vs. GTD). For the substrate data, both the refined SUB and noisy-labeled datasets achieved 100% accuracy when compared with GTD. We then compared the refined datasets (INH and SUB) with their corresponding unmerged counterparts from the noisy-labeled dataset (Fig. 2c). Consistent with the results using GTD, the noisy-labeled dataset displayed a relatively low precision score of 79.7% for inhibitors but a high precision score of 98.1% for non-inhibitors when compared with INH. For the substrate dataset, the noisy-labeled dataset exhibited a high accuracy of 94.5% compared with SUB. These results indicate that our refined datasets serve as high-confidence benchmarks for ABCB1 inhibitor and substrate predictions. Notably, the established INH dataset significantly improved the precision score for inhibitor classification. To ensure fair comparison in subsequent analyses, overlaps between the datasets with different confidence levels were removed from the lower-confidence datasets beforehand.

### MolMM framework for substrate and inhibitor predictions

**Figure 3:**
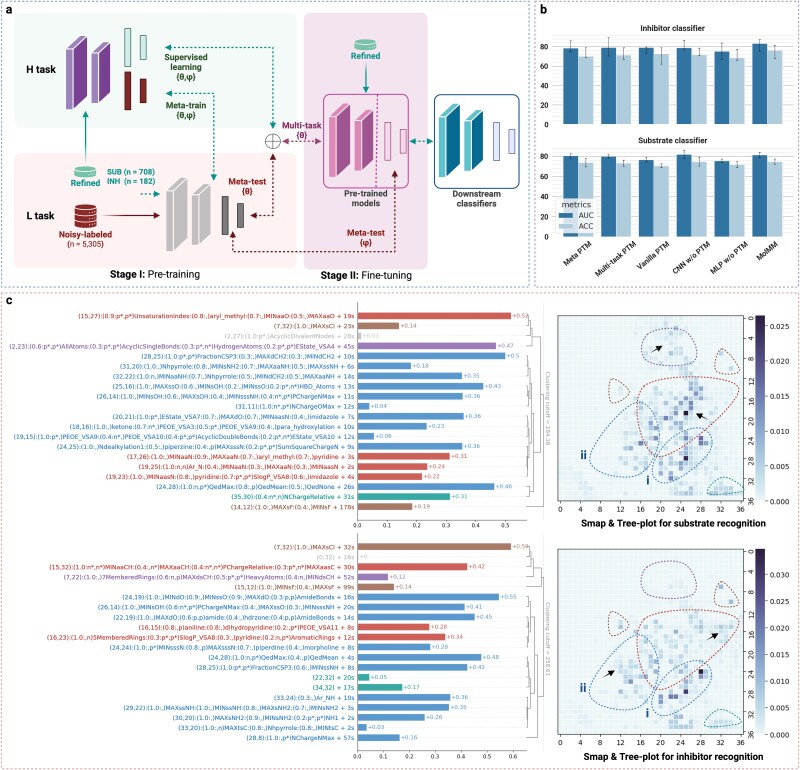
MolMM workflow, model performance, and SHAP analysis for ABCB1 substrate and inhibitor prediction. (a) Overview of the MolMM workflow. Solid arrows indicate data flow, while dotted arrows depict the model’s forward and backward propagation, color-coded by confidence level. For model propagation, updated parameters are annotated on the arrows, where ${{\theta ,\varphi }}$ denote the parameters of shared feature encoders and the task-specific MLP heads, respectively. (b) Comparison of predictive performance for MolMM, benchmark transfer learning models, and vanilla models for inhibitor and substrate predictions. (c) SHAP analysis of MolMM models, visualized using SHAP maps (Smaps) and tree plots. Dashed circles in different colors segment the Smaps and tree plots into clusters according to their semantic significance. Black arrows on the SHAP maps indicate higher-intensity clusters in *INH-* and *SUB-*Smaps. In the tree plots, each bar represents a cluster of molecular descriptors exhibiting high SHAP values. Individual descriptors within each cluster are labeled as “(relative correlation coefficient: m$_{\text{ inh}}$, m$_{\mathrm{sub}}$)”, where the relative correlation coefficient is derived from min–max scaling of SHAP values within the corresponding clusters. Here, m indicates the sign of the rank correlation coefficient, with “p” denoting a positive correlation and “n” a negative correlation. Descriptors are labeled only if the absolute value of the coefficient exceeds 0.8, and those surpassing 0.9 are further marked with an asterisk (“*”).

We first established MolMM framework for these hierarchical-confidence bioactivity data. Specifically, MolMM utilizes a feature encoder established by MolMap method and CNN architecture. We generated the MolMap’s 2D Fmaps for datasets following the standard procedure detailed in the MolMap GitHub repository (https://github.com/shenwanxiang/bidd-molmap) [30]. The 2D Fmaps are obtained by unsupervised learning a structured organization of 1456 molecular descriptors across 8 506 205 molecules. Due to this pretraining step relies solely on unsupervised feature embedding and does not utilize any task-specific labels or downstream target values, it introduces no data leakage into subsequent supervised learning tasks. MolMM’s training comprises two main stages (Fig. 3a): (Stage I) a pretraining phase using a transfer learning framework that leverages both abundant low-confidence samples and scarce high-confidence samples in the bioactivity datasets, and (Stage II) a fine-tuning phase that focuses exclusively on high-confidence samples to optimize model performance. In Stage I, we utilized a multi-task learning framework [31] in MolMM to address two tasks: supervised learning for high-confidence samples (H task) and meta-learning for low-confidence samples (L task) [32]. These two tasks are integrated through shared layers of the MolMM architecture, facilitating the learning of common features across high- and low-confidence samples. For H task, the model is trained directly on the refined dataset. For L task, MolMM conducts meta-training using the refined datasets and meta-testing on the noisy-labeled dataset. Overall, MolMM consists of four key components: (i) a CNN feature encoder, (ii) meta-learning for the L task, (iii) supervised learning for the H task, and (iv) hierarchical-confidence datasets in ML scenarios.

Then, we trained two MolMM models to predict inhibitors and substrates, respectively. For model evaluations, we performed stratified five-fold cross-validation and reported the mean values and standard deviations across all cross-validation test sets to ensure the reliability of evaluation results. The classification performance of models was evaluated using two metrics: area under the receiver operating characteristic curve (AUC-ROC, %) and accuracy (ACC, %). AUC-ROC scores on the test sets of the refined datasets served as the primary metric for comparing classification performance. Figure 3b and Table 1 present the predictive performance of our MolMM models compared with the benchmark transfer learning models using traditional supervised learning, multi-task learning, and meta-learning pretrained models (PTMs) (see ref. [33] for a review of these models, details in SI text section 3A). Furthermore, the models trained via supervised learning on INH and SUB without pretraining are included in the comparison study, specifically CNN w/o PTM and MLP (multilayer perceptron) w/o PTM, which use CNN and MLP architectures, respectively.

**Table 1:** Classification performance of comparison and ablation study for MolMM.

**Models**	**SUB**		**INH**	
	**AUC (%)**	**ACC (%)**	**AUC (%)**	**ACC (%)**
Meta PTM	80.37 (2.23)	73.81 (4.35)	78.45 (5.63)	70.25 (4.89)
Multi-task PTM	79.87 (3.34)	73.10 (3.15)	79.14 (7.73)	71.41 (5.85)
Vanilla PTM	76.47 (1.56)	70.53 (1.77)	79.16 (9.41)	72.71 (8.40)
CNN w/o PTM	81.83 (2.15)	74.67 (4.45)	78.76 (2.87)	71.46 (3.82)
MLP w/o PTM	75.47 (2.29)	71.67 (2.81)	75.31 (3.26)	68.43 (5.86)
MolMM	**81.26 (3.13)**	**74.67 (2.46)**	**83.33 (5.01)**	**76.27 (7.01)**
**Experiment (1)**	**Feature encoders**			
MLP (MolMM)	74.91 (2.86)	73.24 (3.21)	**83.49 (2.91)**	73.80 (1.38)
MLP w/o PTM	75.47 (2.29)	71.67 (2.81)	75.31 (3.26)	68.43 (5.86)
CNN w/o PTM	**81.83 (2.15)**	**74.67 (4.45)**	78.76 (2.87)	71.46 (3.82)
MolMM	81.26 (3.13)	74.67 (2.46)	83.33 (5.01)	**76.27 (7.01)**
**Experiment (2)**	**L task**			
Multi-task PTM	79.87 (3.34)	73.10 (3.15)	79.14 (7.73)	71.41 (5.85)
MolMM	**81.26 (3.13)**	**74.67 (2.46)**	**83.33 (5.01)**	**76.27 (7.01)**
**Experiment (3)**	**H task**			
Meta PTM	80.37 (2.23)	73.81 (4.35)	78.45 (5.63)	70.25 (4.89)
MolMM w/o H	80.47 (1.63)	74.53 (2.48)	78.89 (3.20)	69.66 (3.26)
MolMM	**81.26 (3.13)**	**74.67 (2.46)**	**83.33 (5.01)**	**76.27 (7.01)**
**Experiment (4)**	**Hierarchical-confidence settings**			
Mixed	76.92 (3.47)	72.38 (3.47)	77.20 (9.76)	72.03 (7.32)
MolMM (inverse)	78.82 (2.91)	71.96 (2.05)	77.61 (3.89)	70.27 (3.51)
Multi-task PTM	79.87 (3.34)	73.10 (3.15)	79.14 (7.73)	71.41 (5.85)
MolMM	**81.26 (3.13)**	**74.67 (2.46)**	**83.33 (5.01)**	**76.27 (7.01)**

As a result, our MolMM model demonstrates superior performance on both the inhibitor and substrate predictions. For the inhibitor prediction, the MolMM model outperforms all other models, achieving an AUC-ROC of 83.33$\pm $5.01% and an ACC of 76.27$\pm $7.01%. This represents an improvement of 4.17% in AUC-ROC and 3.56% in ACC compared with the second-best model (vanilla PTM). For the substrate prediction, the MolMM model shows the best performance among all models developed with pre-training methods, with an AUC-ROC of 81.26$\pm $3.13% and an ACC of 74.67$\pm $2.46%. Compared with the approach without pretraining methods, the MolMM demonstrates comparable performance on SUB, with only a slight disparity in $\Delta $AUC-ROC of 0.56% and equivalent ACC on average. For further evaluating MolMM models, we performed a comprehensive ablation study to elucidate the contributions of MolMM’s individual components, as shown in Table 1 (details in SI text section 3B). These results show that explicit hierarchical-confidence settings enhance predictive capability on high-confidence datasets. Moreover, they also highlight MolMM’s capability to transfer information from the L task to the H task within an explicit hierarchical-confidence setting.

### Interpretation of MolMM’s outcomes via SHAP method

We performed a feature importance analysis using SHAP values derived from MolMM models to identify critical molecular features involved in inhibitor and substrate recognition and binding mechanisms [34–37]. Since Fmaps—the input of MolMM—are the grid-like representations derived from 1344 molecular descriptors, the SHAP values for the model can naturally manifest as heatmaps. Here, we refer to these SHAP-derived molecular heatmaps as “SHAP maps.” The 2D SHAP maps are designed to inherit the ability to capture intrinsic correlations among molecular features from Fmaps and CNN architecture, enabling the identification of discriminative clusters of molecular descriptors [30]. These clusters can be intuitively represented by the high-intensity regions on the SHAP maps. We then performed hierarchical clustering analysis to the molecular descriptors based on the image distance on SHAP maps to reveal the details of the discriminative molecular descriptor clusters on the SHAP maps. As a result, the detail clustering results, along with the SHAP values, are visualized as bar plots with tree structures (referred to as “tree plots”). Furthermore, the rank monotonicity between each feature and its SHAP value is quantified using Spearman’s rank correlation coefficient to access the relationship between the feature’s values and the model’s predicted outcomes (see ref. [38] for a review). Figure S2 shows the SHAP dependence plots of critical molecular descriptors discussed in this section, along with their corresponding rank correlation coefficients. Details about generation of SHAP maps and tree plots are provided in the Subsection 4.3. For convenience, we used the prefixes *INH-* and *SUB-* to distinguish the SHAP maps and tree plots from inhibitor and substrate recognition.

First, the red and blue regions in both *INH-* and *SUB-*SHAP maps indicate that the majority of molecular features driving the model’s predictions are associated with polar interaction networks (Fig. 3c). The tree plots for these regions provide insights into the extensive predictive polar interaction sites of molecules, such as hydrogen bond acceptors/donors (HBAs/HBDs), polar bonds, and aromatic systems. Among these features, FractionCSP3—measuring the ratio of $sp^{3}$-hybridized carbons to total carbon atoms—emerges as a critical feature in the blue region of *INH-* and *SUB-*SHAP maps (see ref. [39] for a review). The SHAP dependence plot of FractionCSP3 shows a monotonic increase in its *INH-* and *SUB-*SHAP values with increasing FractionCSP3. Notably, we found a potential competitive relationship between ligand’s polarity and hydrophobicity in both substrate and inhibitor predictions. The unsaturation index serves as a complementary yet competitive molecular descriptor to FractionCSP3, relevant to molecular hydrophobicity, whose *INH-* and *SUB-*SHAP values show a monotonic increase with increasing index value (red region). Moreover, the NChargeRelative index (green region) and PChargeRelative index (red region) quantify the contributions of the most electronegative atom to the molecule’s overall negative and positive partial charges, respectively, contributing to polar interaction networks. Their SHAP dependence plots reveal a monotonic decrease in *INH-* and *SUB-*SHAP values with increasing their feature values, further supporting this competitive relationship.

Then, we examined the differences between the *INH-* and *SUB-*SHAP maps, integrating insights from their SHAP rank monotonicity. Here, the black arrows on the SHAP maps intuitively highlight regions of higher intensity when comparing the *INH-* with the *SUB-*SHAP maps. The arrow in the blue region reveals an expanded high-intensity area in the *INH-*Smap relative to the *SUB-*Smap, corresponding to a cluster of molecular descriptors associated with polar interaction networks. It highlights molecular descriptors relevant for stronger polar interaction networks (such as carboxylic groups, -COO). The arrow in the red and purple region show the differences between *INH-* and *SUB-*SHAP maps among a cluster of molecular descriptors associated with aromatic systems and alkyl . In contrast, inhibitor prediction shows weaker associations with alkyl scaffolds and aromatic heteroatoms (such as pyridine-like aromatic nitrogen). Furthermore, the key findings regarding SHAP’s rank monotonicity are summarized as follows.

- First, the quantitative estimate of drug-likeness (QED [40]), emerges as a critical commonality for inhibitor and substrate recognition (blue region). QED exhibits an inverse impact on substrate and inhibitor predicted outcomes, consistent with pharmacological expectations: inhibitors tend to have higher QED values, whereas substrates generally display lower values.
- Second, *SUB-*SHAP values of key charge descriptors decrease monotonically as their respective values increase, particularly PChargeNMax (maximum positive charge on nitrogen atoms; blue region) and NChargeOMax (maximum negative charge on oxygen atoms; blue region). Conversely, *INH-*SHAP values of these descriptors increase monotonically with their respective values, as does NChargeNMax (maximum negative charge on nitrogen atoms; blue region). These opposing impacts of the charge descriptors on inhibitor and substrate predictions suggest that the inhibitor prediction favors stronger polar interaction networks. Additionally, the quantity of protonated amino groups, also highlighted in the blue region, indicates that cationic motifs in molecules can enhance the inhibitor binding to ABCB1.
- Last, the quantities of HBDs and total hydrogen atoms (blue and purple regions, respectively), as well as the E-state descriptors MAXaasC and MAXaaCH (maximum E-state indexes among substituted and unsubstituted aromatic carbon, respectively [41]; red region) show opposite impacts on substrate and inhibitor prediction. The inhibitor prediction emphasizes hydrogen-deficient motifs and low-polarity aromatic systems compared with the substrate prediction, contributing to the identification of strong hydrophobic motifs that function as hydrophobic anchors. For example, the quantity of aromatic rings—typical hydrophobic anchors—shows that the corresponding *SUB-*SHAP value decreases monotonically with increasing aromatic ring count, while the *INH-*SHAP value demonstrates the opposite trend (red region).

### Calculation of potential of mean force profiles for molecules based on the biological insights

**Figure 4:**
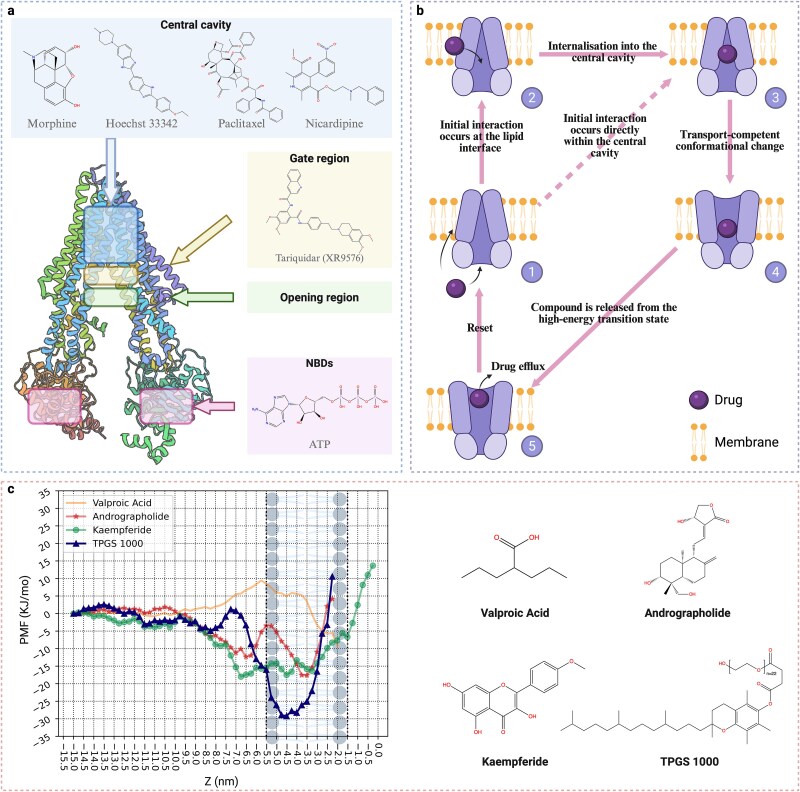
Mechanistic insights into ABCB1 allocrite binding and inhibition from MD simulations. (a and b) Schematic overview showing (a) critical regions within the ABCB1’s transmembrane pore, and (b) two binding pathway of allocrites to ABCB1: (1) the membrane pathway: 1 $\to $ 2 $\to $ 3 $\to $ 4 $\to $ 5; (2) the transmembrane-pore pathway: 1 $\to $ 3 $\to $ 4 $\to $ 5. (c) PMF profiles for representative allocrites, illustrating free energy changes along the binding pathway from the aqueous phase into the ABCB1 central cavity through transmembrane pore, and highlighting specific energy barriers and minima.

PMF profiles characterize the energy landscape of allocrite binding pathways, tracing the movement from the aqueous phase to the central cavity through the transmembrane pore (referred to as PMF$_{\mathrm{pore}}$). For this purpose, we adapted the strategy established by Subramanian *et al*. [19, 20]. Specifically, we employed a coarse-grained ABCB1 model (PDB: 4M1M) embedded in a pure POPC bilayer, rather than the original all-atom ABCB1 model (PDB: 3G5U) in a 9:1 POPC and cholesterol bilayer. This modification required recalibration of reaction coordinates to account for lateral deviations between membrane-embedded ABCB1 models (Fig. 4a). To enable comparisons of PMF profiles, we assigned the origin of PMF profiles ($Z^{\prime} = 0$, i.e. $Z = -3.5$ nm as shown in Fig. 4c) at the membrane’s hydrophobic core region (details of the settings of MD simulations refer to SI text section 4).

Valproic acid, a short branched fatty acid, has been reported as a non-substrate for ABCB1 [42]. Since the molecular descriptor cluster associated with polar interaction networks are considered as the driving factor for substrate and inhibitor predictions in MolMM models, we used neutral valproic acid as a probe to identify probable polar interactions on ABCB1’s transmembrane pore through its PMF profile. To explore the substrate binding pathway, we calculated the profile of hydrophobic terpenoid andrographolide, a small-molecule ABCB1 substrate [43, 44] and kaempferide. Notably, kaempferide shares a similar flavonoid backbone with kaempferol, differing only by an additional methoxy group at the 4’ position. Since kaempferol is known to be transported by ABCB1 [45], this structural similarity suggests that kaempferide may also function as a substrate. Since, kaempferide has additionally been reported to inhibit ABCB1-mediated drug efflux [46–48], we also used its PMF profile to explore the inhibitor binding pathway. Furthermore, TPGS 1000, a polymeric pharmaceutical excipient considered safe and inactive, has nonetheless been shown to inhibit ABCB1 [43, 49]. With approximately a four-fold greater molecular weight compared with the small-molecule kaempferide, TPGS 1000 was selected to further elucidate inhibitor binding pathways through its PMF profile, highlighting size-dependent interactions in ABCB1’s polyspecific recognition. Key observations are as follows.

- First, PMF$_{\mathrm{pore}}$ profile of valproic acid indicates an increase in energy as it approaches the transmembrane opening $Z^{\prime} \approx -2.0$ nm from the aqueous phase, corresponding to the membrane’s lipid–water interface and ABCB1’s transmembrane opening, where the energy peaks. Subsequently, the profile displays a barrier hump at $Z^{\prime} \approx -2.0$ nm, followed by a decline to a minimum at $Z^{\prime} \approx -1.0$ nm, corresponding to the membrane’s headgroup-alkyl border and the cavity gate region. As valproic acid moves further into the cavity, the profile exhibits another decrease near $Z^{\prime} \approx 0$, corresponding to the membrane’s hydrophobic core and the cavity apex. Additionally, we compared the PMF$_{\mathrm{pore}}$ profile of valproic acid with its PMF profile for permeation through a pure lipid bilayer consisting of dipalmitoylphosphatidylcholine (DPPC) (denoted as PMF$_{\mathrm{dppc}}$) [50], aiming to explore the energy minima and barriers along the transmembrane pathway, with and without the influence of the transmembrane pore. As a result, both PMF profiles exhibit similar energy variations from the aqueous phase to $Z^{\prime} \approx -1.0$ nm and a significant divergence emerging as valproic acid penetrates deeper toward $Z^{\prime} \approx 0$: PMF$_{\mathrm{dppc}}$ reaches a minimum at $Z^{\prime} \approx -1.0$ nm and then steadily increases to a maximum at $Z^{\prime} \approx 0$, while PMF$_{\mathrm{pore}}$ drops in this region.
- Second, both andrographolide and kaempferide exhibit similar energy barriers at the transmembrane opening ($Z^{\prime} \approx -2.0$ nm) and energy minima at the cavity apex ($Z^{\prime} \approx 0$) on PMF$_{\mathrm{pore}}$ profiles as valproic acid. The ranking of these energy barriers follows the order: kaempferide < andrographolide < 0 < valproic acid.
- Last, PMF$_{\mathrm{pore}}$ profiles of both kaempferide and TPGS show energy minima at the cavity gate region ($Z^{\prime} \approx -1.0$ nm). In contrast, the substrate andrographolide displays an energetically favorable pathway from the transmembrane opening, with a single minimum inside the cavity and no minimum at the gate region.

### Transport and inhibitory models of ABCB1 allocrite binding

We propose an amphiphilic model for substrate binding to ABCB1, wherein allocrite entry into the central cavity via the transmembrane pore resembles a flip–flop process typical of amphiphiles, governed by a balance of hydrophobic interactions and polar interaction networks. First, for polar interaction networks, the difference between PMF$_{\mathrm{pore}}$ and PMF$_{\mathrm{dppc}}$ profiles for valproic acid at cavity apex suggests favorable polar interactions that stabilize an energy minimum in the PMF$_{\mathrm{pore}}$ within this region. We thus posited that polar interaction networks at the cavity apex represent transport-competent contacts for ABCB1, as evidenced by the deep energy minima observed for substrates like andrographolide and kaempferide. These biophysical findings from MD-derived PMF profiles align with SHAP analyses of the MolMM model for both substrate and inhibitor predictions, which underscore the critical role of polar interaction networks in driving allocrite recognition and binding.

Furthermore, the free energy barriers at the transmembrane pore opening, as observed in the PMF$_{\mathrm{pore}}$ profiles of valproic acid, andrographolide, and kaempferide, likely represent critical barriers that restrict polar contacts between allocrites and the cavity’s residues. These barriers elucidate why valproic acid functions as a non-substrate, despite its demonstrated capacity for polar interaction in the PMF$_{\mathrm{pore}}$ profile. In contrast to substrates like andrographolide and kaempferide, this restriction may arise from high desolvation penalties associated with polar motifs of valproic acid. Thus, the binding process would require an amphiphilic nature in allocrites, interacting with the polar cavity’s residues while overcoming desolvation penalties at the transmembrane pore opening. This mechanism can elucidate the competitive interplay between molecular polarity and hydrophobicity in driving substrate and inhibitor prediction from SHAP analyses in MolMM models. As a result, we propose an amphiphilic region localized at the cavity gate region, whose hydrophobic region extends from the transmembrane opening to the gate region while the polar cavity apex is considered as its hydrophilic region.

Our SHAP analysis for inhibitor prediction suggests that the molecular basis of inhibition lies in the amphiphilic nature conferred by strong polar and hydrophobic motifs. Thus, building on the amphiphilic model, we speculated that the cavity gate region serves as an inhibitory site, as indicated by the energy minima, observed in PMF$_{\mathrm{pore}}$ profiles of kaempferide and TPGS in this region. This inhibitory site suggests an inhibitory model in which inhibition is driven by strong amphiphilic interactions. Specifically, the polar regions of inhibitors might interact with the cavity through polar interaction networks, while their hydrophobic regions bind to the expected hydrophobic region of the cavity gate region strongly This formation of strong polar interaction networks reduces the flexibility of the cavity, which, together with the hydrophobic anchors remaining outside the cavity, synergistically stabilizes the ABCB1-inhibitor complex, thereby forming a metastable state of the complex’s conformation. This inhibitory model suggests that inhibitors trap ABCB1 in a conformational transition state (the transition from conformation 1 $\to $ 3 in Fig. 4b), thereby interfering with the conformational dynamics necessary for substrate transport (the transition from conformation 3 $\to $ 4 in Fig. 4b) and thus cutting off the substrate transport cycle.

## Discussion

In this study, our MolMM model surpasses benchmark transfer learning approaches, highlighting MolMM’s efficacy in handling data heterogeneity and noisy labels inherent to multi-source ABCB1 functional assays. SHAP analysis of MolMM revealed discriminative molecular features, emphasizing competitive polar, and hydrophobic motifs that distinguish substrates from inhibitors. Polar interaction networks emerged as primary drivers for both substrate and inhibitor predictions, with high-intensity regions in SHAP maps indicating cohesive clusters of descriptors related to hydrogen bonding, electrostatic potential, and polar surface area. Hydrophobic motifs, including aromatic ring counts and unsaturated index, showed variant influences: positively correlating with substrate prediction but often exerting opposing effects in inhibitor prediction, as evidenced by rank monotonicity calculations between descriptor values and predicted outcomes.

Building on these ML-derived insights, coarse-grained umbrella sampling MD simulations mapped these features onto free energy landscapes for representative allocrites: valproic acid (non-substrate), andrographolide (substrate), kaempferide (substrate with inhibitory potential), and TPGS 1000 (inhibitor). The PMF profiles delineated binding pathways from the aqueous phase through the transmembrane pore, revealing metastable states and energy barriers aligned with SHAP-identified motifs. For substrates like andrographolide and kaempferide, low energy barriers at the transmembrane opening and deep minima within the central cavity underscored an amphiphilic model of binding, wherein polar motifs facilitate entry via a flip–flop process, complemented by hydrophobic interactions fully entering the transport-competent sites. In contrast, inhibitors such as TPGS 1000 and kaempferide exhibited energy minima at the cavity gate region, suggesting a mechanism that arrests ABCB1 in transitional states, preventing ATP-driven conformational shifts essential for efflux. Non-substrates like valproic acid faced high desolvation penalties and barriers, restricting polar contacts despite favorable interactions at the cavity apex.

Notably, some previously published results support our ABCB1 transport and inhibitory models (details in SI text section 5). As a result, this MD-ML synergy not only generalizes biophysical insights across diverse chemical spaces but also provides a unified mechanistic view of ABCB1 polyspecificity, where molecular properties drive dynamic binding processes unachievable by either method in isolation.

## Materials and methods

### Data collation and cleaning

The screening strategy for establishing the hierarchical-confidence datasets followed a structured workflow comprising three main steps: data collation, cleaning, and aggregation (Fig. 2a).

First of all, Table S1 summarizes the collected inhibition and efflux assay records from public databases or published literature. We classified the data into two groups: annotated records containing both experimental metadata and results, and unannotated records with only classification results but no experimental metadata. The multi-source datasets underwent rigorous preprocessing through three sequential steps using the RDKit toolkit [51]: first, we removed inorganic compounds and metal complexes; second, pharmaceutical salts were neutralized to their corresponding free acid/base forms; and third, all molecules were standardized to canonical SMILES strings to ensure structural consistency.

For establishing the refined datasets, we retained only those annotated records that met the experimental metadata criteria specified in Table S2. First, we stratified these assay records by experimental metadata, and grouped those with measurements conducted under similar conditions. Inhibition and efflux assay records were systematically organized into hierarchical trees. Specifically, inhibition assay records were structured as three-level hierarchies: “SMILES string ^*^ probe substrate ^*^ cell line,” while efflux assay records were organized as two-level hierarchies: “SMILES string ^*^ cell line.” Notably, for efflux assay records, each hierarchy contained only a single node, reducing the tree structure to a linear form. Second, we utilized IC$_{50}$/K$_{i}$ values for inhibitors and efflux ratios (ER) for substrates as the measured metrics, respectively [29, 52]. Based on inhibitory activity, we classified compounds as: inhibitors (IC$_{50}$  $\leq $ 15 $\mu $ M), non-inhibitors (IC$_{50}$  $\ge $ 50 $\mu $ M), and ambiguous inhibitors (15 < IC$_{50}$ < 50 $\mu $ M). To account for the boundary condition of inhibitor identification in competitive inhibition kinetics, the assumption that substrate and inhibitor concentration is equal to IC$_{50}$ and K$_{m}$, respectively, provides the relationship of IC$_{50}$ = 2$\cdot $Ki [53]. Considering transporter substrate potential, compounds with ER > 2 were labeled as substrates, and those with ER $\leq $ 2 were labeled as non-substrates. Then, we removed the duplicates and records with conflicting labels in each leaf node in hierarchical trees. These initial steps of data collation and cleaning resulted in 708 *in vitro* efflux assay records related to 708 test compounds, 306 *in vitro* inhibition assay records for 185 test compounds, and 54 *in vivo* inhibition assay records for 54 test compounds.

Then, the bottom-up data aggregation strategy for bioactivity data is crucial for addressing the heterogeneity in bioactivity labels arising from varying experimental conditions. Since *in vitro* efflux assay data and *in vitro* inhibition assay data had consistent experimental conditions, they did not require data aggregation. For the *in vitro* inhibition assay data, aggregation was performed from cell line nodes up to probe substrate nodes, with SMILES strings of the test compounds at the top level. There were three labels assigned to the nodes: strong inhibitor, moderate inhibitor, and non-inhibitor labels, representing different levels of inhibitory potency. For node aggregation, we applied a majority voting rule to resolve data conflicts. Specifically, if a node had only strong and moderate inhibitor labels, we compared the frequencies of each: if the strong inhibitor labels surpassed the moderate ones, the node was assigned the inhibitor label; if a node had only non-inhibitor labels, it was assigned the non-inhibitor label; otherwise, it was excluded from the dataset, ensuring robustness for inhibitor labels across different experimental conditions. Subsequently, we constructed a noisy-labeled dataset from residual assay data excluded during the screening process, mainly consisting of records lacking key experimental metadata. Unlike the refined datasets, this dataset mixed all substrate/non-substrate and inhibitor/non-inhibitor assay data to categorize compounds into two broad classes: allocrites (compounds interacting with the transporter as either substrates or inhibitors) and non-allocrites.

### MolMM’s data flows

Details of MolMM’s deep learning architecture and hyperparameters are shown in Supplementary text section 2. Here, we focused on data flows of MolMM. When working with a batch of SMILES data $s_{n}$ of size $N$ sampled equally from both the noisy-labeled dataset and the refined dataset, the corresponding molecular representations $d_{\mathrm{noisy}}$ and $d_{\mathrm{refined}}$ are constructed using molecular descriptors, such as 1D tabular descriptors or 2D MolMap Fmaps. These two subsets are processed through a shared feature encoder $f(d;\theta )$, mapped into latent representations $h_{\mathrm{noisy}}$ and $h_{\mathrm{refined}}$. Task-specific MLP heads $g(h;\varphi )$ then take these latent representations and outputs classification results, $z_{\mathrm{noisy}}$ and $z_{\mathrm{refined}}$, parameterized separately by $\{{\varphi }_{H},\varphi _{L}\}$, respectively. To address potential conflicts arising from the multi-task learning setup, gradients from the H and L tasks are balanced using the Multiple Gradient Descent Algorithm Upper Bound (MGDA-UB), $m(\{\cdot \})$. This approach ensures a Pareto-optimal trade-off between the update gradients calculated for the two tasks during training [54]. It is important to highlight that the PTM’s parameters, used for downstream fine-tuning, correspond to the parameters of the L task, namely $\{\theta ,\varphi _{L}\}$. For our approach, the loss functions cross-entropy loss, $\mathcal{L}(\cdot )$ associated with meta-learning and multi-task learning tasks are leveraged to compute the gradient updates for parameters $\{\theta ,\varphi \}$ by Equations 1,3, where $\alpha $ and $\beta $ are the hyperparameters for the training processes of meta-learning and multi-task learning, respectively. 


(1) \begin{align*} &\mathrm{grad}_{\text{L task}}(\theta,{\varphi}_{\text{L task}})=\nabla \mathcal{L}(z_{\mathrm{noisy};~\theta^\prime,{\varphi^\prime}_{\text{L task}}}); \nonumber\\ &(\theta^\prime,{\varphi^\prime}_{\text{L task}})=(\theta,{\varphi}_{\text{L task}})-\alpha\cdot \nabla \mathcal{L}(z_{\mathrm{refine};~\theta,{\varphi}_{\text{L task}}}) \end{align*}

(2) \begin{align*} &\mathrm{grad}_{\text{H task}}(\theta,{\varphi}_{\text{H task}})=\nabla \mathcal{L}(z_{\mathrm{refine};~\theta,{\varphi}_{\text{H task}}}) \nonumber\\ &\mathrm{grad}_{\mathrm{update}}(\theta)=\mathrm{grad}_{\text{H task}}(\theta)+\beta\cdot\mathrm{grad}_{\text{L task}}(\theta);\nonumber\\ &\beta=m(\{\mathrm{grad}_{\text{H task}}(\theta),~\mathrm{grad}_{\text{L task}}\left(\theta\right)\}) \end{align*}

(3) \begin{align*} &\mathrm{grad}_{\mathrm{update}}(\varphi)=\{\mathrm{grad}_{\text{H task}}(\varphi),~\mathrm{grad}_{\text{L task}}(\varphi)\} \end{align*}

### Generation of SHAP maps and tree plots

MolMM’s 2D Fmaps, used as the input for MolMM, are essentially ordered tabular molecular descriptors presented as 2D grid images. Consequently, each pixel on Fmaps corresponds to a meaningful molecular descriptor and correlates with those of the nearby pixels. We can intuitively explain CNN-based models in a similar way to conventional MLP models that use 1D tabular molecular descriptors. Fmaps consist of 1344 molecular descriptors in 13 categories (Property, Constitution, Fragment, Charge, Estate, MOE, Topology, Kappa, Connectivity, Autocorr, Path, Matrix, and InfoContent), where descriptors belonging to the first eight categories are considered intuitively interpretable features (see ref. [35] for a review). Compared with MLP models, CNNs preserve the spatial information of 2D grid data, and thus their SHAP values reflect this inherent dependency structure. 


(4) \begin{align*} \mathrm{SHAP}^\prime(i)=\mathrm{SHAP}(i)+\sum_{d_{i,j}\le c;\ i\neq j}\frac{\mathrm{SHAP}(i)-\mathrm{SHAP}(j)}{\alpha\cdot d_{i,j}^{2}}\end{align*}

Here, we utilized DeepExplainer from the SHAP package [37] to compute SHAP values of 2D Fmaps to generate the SHAP maps. Subsequently, we derived insights into the crucial molecular features of substrate and inhibitor recognition from ML models by identifying and analyzing the local high-intensity regions of SHAP maps. Notably, we employed relative SHAP values instead of the original ones, filtering out relatively lower SHAP features in local high-intensity regions of SHAP maps and emphasizing those with relatively higher SHAP values in low-intensity regions. The variants of SHAP values are shown in Equation (4), whose sum remains consistent with the original, $\alpha $ is a hyperparameter that quantifies how image distance impact the SHAP values on SHAP maps, and $c$ represents the cutoff distance. We then applied the hierarchical clustering method on SHAP maps, creating bar plots with a clustering tree structure based on molecular features and variant SHAP value pairs, referred to as Smap tree plots. The clustering structure was determined using the pixel-pair distance matrix on SHAP maps, $d(\cdot ,\cdot )$. This is intended to capture the detailed molecular features in each critical cluster on SHAP maps. Consequently, high-intensity regions on SHAP maps are represented by groups of spatially adjacent molecular features within a cutoff distance $c$ from features with local maximum variant SHAP values, displayed on tree plots. The features of these groups are sorted by their variant SHAP values in descending order.

**Key Points**

- The integrated framework synergistically combines machine learning and molecular dynamics to predict ABCB1 substrates and inhibitors while elucidating their interaction mechanisms, addressing limitations in experimental approaches.
- Hierarchical-confidence bioactivity datasets were curated from multi-source assays, enabling the development of the MolMM convolutional neural network, which achieved AUC-ROC scores of 83.33% for inhibitors and 81.26% for substrates through meta-learning on noisy data and multi-task learning on refined data.
- SHAP analysis revealed discriminative molecular features, including competitive polar and hydrophobic motifs that distinguish substrates from inhibitors, with polar interactions favoring recognition and hydrophobicity influencing binding dynamics.
- Coarse-grained umbrella sampling simulations generated potential of mean force profiles, mapping these features onto free energy landscapes to propose an amphiphilic model for substrate binding via a flip–flop process and an inhibitory mechanism that stabilizes transitional ABCB1 conformations.

## Supplementary Material

Supplementary_Information_bbag106

## Data Availability

The raw data, codes, and parameters of this work is available in the GitHub repository (https://github.com/PatrickSu1101/MolMM). This repository provides: (i) scripts for development and preprocessing of hierarchical-confidence ABCB1 bioactivity datasets, whose original bioactivity data can be found in the repository, (ii) codes for model implementation, including MolMM, benchmark, and ablation study models, (iii) codes for feature embeddings of MolMM models, (iv) codes for SHAP analysis of MolMM models (generating “Smaps” and “tree plots”), and (v) topology parameters for coarse-grain MD simulation (membrane-embedded ABCB1, valproic acid, andrographolide, kaempferide, and TPGS 1000) and their PMF profiles.
