# Supplementary material for: Integrated machine learning and molecular dynamics framework for predicting and elucidating ABCB1 allocrite interactions
Source: Brief Bioinform. 2026 Apr 7;27(2):bbag106. doi: 10.1093/bib/bbag106 (PMC13064662; doi:10.1093/bib/bbag106)
Supplement: Supplementary_Information_bbag106 [file supplementary_information_bbag106.pdf]

Supporting Information for

Integrated Machine Learning and Molecular Dynamics Framework for Predicting and Elucidating ABCB1 Allocrite Interactions

Jianjia Su, Yiyang Wu, Wei Xiong and Defang Ouyang

Wei Xiong & Defang Ouyang
E-mail: weixiongszu.edu.cn (Xiong); defangouyang@um.edu.mo (Ouyang)

This PDF file includes:

Supporting text
Fig. S1
Tables S1 to S3
SI References

Supporting Information Text

1. Recent machine learning models for ABCB1 bioactivity predictions

A. Recent ligand-based machine learning models for predicting ABCB1 substrates and inhibitors. A variety of traditional machine learning methods, such as random forest (RF), support vector machine (SVM), and kappa nearest neighbor (KNN), have been used to classify compounds as ABCB1 substrates versus non-substrates or inhibitors versus non-inhibitors. These models have demonstrated promising accuracy, achieving 67-70% for substrates and over 70% for inhibitors on external test sets (1–3). Furthermore, advanced deep learning models also have been developed for these tasks, achieving even higher predictive performance (4).

B. Overview of ABCB1 *In vitro* functional studies. *In vitro* ABCB1-mediated efflux assays are widely used experimental approaches to evaluate the transporter's functional activity and to characterize the interaction of compounds with ABCB1(5). These assays typically employ cultured cell lines or membrane vesicles engineered to overexpress ABCB1 , and use fluorescent or radiolabeled substrates known to be transported by ABCB1. The extent of substrate efflux from cells or vesicles serves as a direct measure of ABCB1 activity. By comparing substrate accumulation or efflux in the presence and absence of test compounds or known inhibitors, these assays can determine whether a compound is a substrate or modulator of ABCB1.

In vitro ABCB1 inhibition assays are widely employed to evaluate the potential of new chemical entities and existing drugs to inhibit ABCB1 function(6). These assays typically utilize cell lines or membrane vesicles overexpressing ABCB1 and measure the accumulation or efflux of specific substrates in the presence or absence of test compounds. The degree of ABCB1 inhibition is quantified by comparing substrate transport in the presence of the test compound to that observed with known ABCB1 inhibitors or vehicle controls. Common readouts include fluorescence or radiolabel measurements, depending on the substrate used. The collected *in vitro* assay records are shown in Table S1, and their critical experimental metadata is summarized in Table S2.

C. Overview of data bottleneck for ABCB1 inhibitor data. The variations of ABCB1 inhibition assays were primarily attributed to two experimental factors: probe substrates and inter-laboratory variability(7, 8). It is has been reported that ABCB1 inhibition exhibited substrate-independent behavior for CsA, elacridar, and quinine, exhibited minimal variation across probe substrates. In contrast, quinidine, azole antifungals (ketoconazole, itraconazole), and verapamil demonstrated substrate-specific inhibition patterns, particularly affecting BODIPY-verapamil/prazosin efflux with marked IC_{50} variations ($>$ 2-fold decrease or $<$ 0.5-fold increase) between probe substrates(9). Meanwhile, significant inter-laboratory inconsistencies emerged in cell/vesicle-based IC_{50} measurements, where 13 of the 15 tested inhibitors exhibited 20-fold variability between the minimum and maximum recorded values, while only two compounds demonstrated relatively stable measurements below this threshold(8).

2. The settings of MolMM models

A. MolMM's deep learning architecture. The architecture design of the CNN extractor in MolMM is based on the MolMapNet-D architecture (10). The first convolutional layer of the extractor is equipped with a larger number of kernels (48) of size 13×13. After each convolution layer, a max-pooling layer (3×3) with a stride of 2 is applied. The CNN extractor integrates a naive inception layer inspired by GoogLeNet (11), which incorporates three parallel small kernels with dimensions 1×1, 3×3, and 5×5. Within the extractor, there are two naive inception layers featuring kernels of sizes 32×3 and 64×3, respectively. A global max-pooling layer is included to minimize parameters and interface with a single hidden layer containing kernels of size 64. These layers serve as shared layers in a multi-task learning framework. Following these shared layers are task-specific MLP heads, each consisting of three hidden layers with kernel sizes of 64, 32, and 2, respectively. For MLP architecture design, it includes a shared feature extractor with three linear layers (input: 1344 to 128, followed by two 128-to-128 layers, each with ReLU activations) and task-specific branches: dimensionality-reducing modules (128 to 64 to 32) per task.

B. MolMM's hyperparameters. We utilized MolMM deep learning models with ReLU activation functions. The optimization process employed the AdamW optimizer to update gradients (12), calculated using cross-entropy loss. The original training dataset, obtained through cross-validation splitting, was further divided into a training set and a validation set. The proportion of the validation set was determined using Equation 1 (13). An initial outer learning rate of 1×10^{-3} was applied, along with a learning rate decay schedule that reduced the rate when the cross-entropy loss on the training set stopped improving. This schedule included a patience value of 10 and a decay factor of 0.5. The model was trained with an overall batch size of 32 and 4 shots per batch. To manage the training duration, we applied an early stopping technique that halted the process when validation set accuracy ceased improving, with a patience of 40 epochs and a maximum limit of 500 epochs (14). For downstream fine-tuning, additional training was conducted on the pre-trained model. During this phase, the batch size remained set to 32, and early stopping was again applied, with the same criteria of 40 patience epochs and a maximum of 200 epochs to achieve optimal validation set accuracy. Both MolMM implementation and fine-tuning were performed using PyTorch platform (15).

$$\text{Split ratio} = \frac{1}{\text{training set size}^{0.25} + 1} \qquad [1]$$

3. The settings of MolMM comparative analysis

To ensure consistency in the comparative analysis, all models employed shared the same framework and hyperparameters as the MolMM models, with the sole difference being the activation or deactivation of various components.

A. Comparative analysis of the benchmark transfer learning models.

- Vanilla PTM: This baseline model is directly supervised learned on the noisy-labeled dataset without the noise mitigation.
- Multi-task PTM: This baseline employs a multi-task learning framework within the pre-training stage, noisy-labeled and refined datasets are learned simultaneously to identify shared latent features.
- Meta PTM: This baseline utilizes a meta-learning approach acquiring generalizable strategies from noisy-labeled and refined datasets to improve the adapt efficiency.

B. Ablation analysis of MolMM components.

- Experiment (1): We compared CNN and MLP architectures as feature encoders to assess the impact of these encoders on model performance. For supervised learning models, the CNN significantly outperformed the MLP, achieving improvements in ΔAUC-ROC (INH: 3.45%; SUB: 6.36%). For MolMM, CNN maintains a ΔAUC-ROC of 6.35% over MLP in substrate prediction, while the improvement is not significant for MolMM in inhibitor prediction.
- Experiment (2): To evaluate the meta-learning component for the L task, we compared MolMM to multi-task PTM, a variant of MolMM in which the meta-learning framework is replaced with supervised learning for the L task. MolMM demonstrated significant improvements in ΔAUC-ROC values over multi-task PTM (INH: 4.19%; SUB: 1.39%).
- Experiment (3): To assess the supervised learning component for the H task, we generated a variant by removing the H task from MolMM, retaining only the meta-learning component for the L task (referred to as MolMM w/o H). We then compared MolMM with MolMM w/o H and meta PTM. MolMM demonstrated superior AUC-ROC performance on both INH and SUB, outperforming MolMM w/o H and meta PTM. The ranking of models by AUC-ROC was: MolMM > MolMM w/o H > meta PTM. The performance gap between MolMM and MolMM w/o H reached ΔAUC-ROC values (INH: 4.44%; SUB: 0.79%).
- Experiment (4): To analyze the effect of the hierarchical-confidence setting for training datasets, we compared MolMM to two variant transfer learning models: the inverse model, in which data flows of refined and noisy-labeled datasets are exchanged during training, and the mixed model, which is trained on all data from refined and noisy-labeled datasets based on the supervised learning. MolMM achieved superior AUC-ROC scores on both INH and SUB. The ranking of models by AUC-ROC was: MolMM > multi-task PTM > inverse model > mixed model.

4. The settings of coarse-grained umbrella sampling methods

A. Umbrella sampling and window setup. Umbrella sampling and window setup were carried out using a strategy adapted from the methodology outlined by Subramanian et al.'s work (16, 17). The initial configuration for all MD simulations in this study was derived from a pre-equilibrated conformation of the ABCB1 protein (PDB code: 4M1M), embedded within a pure POPC lipid bilayer and solvated with water containing 0.15 M NaCl. This configuration was generated using the Martini Bilayer Maker tool available in the CHARMM-GUI package (18, 19). The protein and lipid coordinates were sourced from the Orientations of Proteins in Membranes database (20). Component parameters for the system were defined based on the Martini 3.0 force field (21). All MD simulations were executed using OpenMM version 3.1.0 (22), augmented by toolkits from the GROMACS package (23). Simulations were conducted under periodic boundary conditions within a rectangular box measuring 13 nm × 13 nm × 18 nm. A reaction field correction was applied with a relative dielectric constant (ϵ_r) of 15.0 to reduce artifacts caused by truncating electrostatic interactions beyond the 1.1 nm cutoff distance. To accommodate the anisotropic nature of the bilayer, a barostat was employed to decouple pressure along the bilayer plane (X and Y axes) from the vertical (Z axis). The simulations were run in the NPT ensemble at a temperature of 300 K and a pressure of 1 bar, using a coarse-grained time step of 20 fs. Data collection for the collective variable in each simulation window occurred every 20 ps for subsequent analysis.

In the umbrella sampling setup, the reaction coordinate axis is aligned perpendicularly to the lipid bilayer and passes through the center of mass (COM) of the reference group, defined as the apex of ABCB1 (16, 17). The reference group's reaction coordinate is set at $Z = 0$. The collective variable was defined as the position on the Z-axis corresponding to the COM of the ligand. For each ligand, 60 umbrella sampling windows were generated. The final window was established with the ligand positioned in the aqueous phase at $Z = -15\ nm$. From there, the ligand was moved incrementally along the reaction coordinate axis, creating sampling windows at intervals of 2.5 Å from the origin to the final window. Each window included a harmonic potential applied between the ligand and protein, determined by the Z-direction distance between their respective COMs. The force constant for this restraint was set at 500 $kJ/(mol \cdot nm^2)$. A 100-ns extended MD simulation was conducted for each window, yielding 5,000 frames of reaction coordinate data per ligand in every window. To ensure convergence, it is noteworthy that Subramanian et al. (16, 17) had previously suggested extended simulation times of 50-60 ns per window; hence, the 100-ns simulations performed in this study were deemed sufficient for achieving convergence. Following these simulations, each ligand's PMF profile was calculated using the weighted histogram analysis method.

B. Coarse-grained MD parameters of ABCB1 allocrites. All-atom MD parameters for kaempferide (PubChem 5281666), andrographolide (PubChem 5318517), valproic acid (PubChem 3121), and TPGS (PubChem 71406) were defined using the ACPYPE Server (24). At pH 7.4, the protonation state of each molecule is neutral except for valproic acid; however, we used valproic acid in its neutral form here. For TPGS, due to its large molecular mass, we specifically calculated the parameters for its monomer before extending them to the complete structure, ensuring parameter accuracy. The ACPYPE tool provided GAFF parameters (25), derived from partial charges calculated using BCC method (26). Coarse-grained parameters were developed following the strategy (27). These parameters for bond lengths, angles, and dihedral angles were extrapolated from MD simulation trajectories corresponding to their all-atom models. Here, we only compared the coarse-grained PMF_{pore} profiles with all-atom profiles previous studies to validate our coarse-grained models, since our primary objective was to identify specific energy barriers and minima in the PMF profiles instead of quantitative analysis for those PMF profiles.

5. Comparison with previously published results for our ABCB1 transport and inhibitory models

First, previous structural studies by cryo-EM techniques have demonstrated the ordered cholesterol-phospholipid complexes bound to ABCB1's exterior transmembrane helices (28). When combined with the proposed amphiphilic region within the transmembrane pore, this finding suggests an opposite arrangement of amphiphilic regions: one in the transmembrane pore and the other at the ABCB1's lipid interface. This arrangement might serve a dual functions: (1) it stabilizes ABCB1 within the lipid environment via amphiphilic interactions between the lipid interface and the lipid bilayer, due to the consistent arrangement of polarity; (2) it generates energy barriers that prevent components of lipid bilayer from entering the transmembrane pore due to the opposite amphiphilic nature of the pore. Additionally, the hydrophobic region proposed in our amphiphilic model is supported directly by ABCB1's structural results. Nosol et al. identified a phenylalanine-rich cavity, referred to as the "access tunnel" in the cryo-EM structure of inhibitor-bound ABCB1, into which one of the paired inhibitors extends (29). Interestingly, the "access tunnel" is located at the border between the cavity and the transmembrane opening, a location that aligns with the cavity gate region along the Z-axis in our inhibitory model.

Then, our analysis of all-atom PMF_{pore} profiles from Subramanian et al. (morphine, Hoechst 33342, paclitaxel, nicardipine, rhodamine 123, tariquidar, and verapamil) reveals similar energy minima and barriers to our PMF profiles (16, 17). First, for energy minima, all these allocrites exhibit energy minima within the central cavity, consistent with their potential to be transported by ABCB1. Second, for energy barriers, Rhodamine 123, nicardipine, and paclitaxel exhibit energy barriers near the cavity gate region. Hoechst 33342 follows the PMF_{pore} profile of andrographolide, with a barrier near the transmembrane opening and an energetically favorable pathway toward the cavity's minimum. In contrast, the competitive binding pair of morphine and verapamil (30) show overall descending PMF_{pore} values as the pair approach the cavity from an aqueous phase, forming energy troughs within the cavity without energy barriers across the transmembrane pore. Notably, steric effects might also emerge as critical the allocrite binding to ABCB1, as indicated by insights from all-atom PMF_{pore} profiles. For example, globular and rigid molecules with polycyclic structures—such as rhodamine 123, nicardipine, and paclitaxel—demonstrate energy barriers near the narrow gate region of the cavity ($Z' \approx -1.0\ nm$), rather than at the transmembrane opening ($Z' \approx -2.0\ nm$). These barriers also emerge in our PMF_{pore} profile of macromolecular TPGS, in which a rapid surge of energy occurs upon approaching the gate region, suggesting that they might arise from steric clashes between allocrites and the cavity gate region. Collectively, these shared free energy landscape features validate the utility of coarse-grained PMF_{pore} calculations in biophysical modeling, enabling the efficient capture of allocrite binding dynamics including energy minima within cavity and barriers at formed by hydrophobic regions.

Last, distinct inhibitor-specific energy barriers and minima observed in all-atom PMF_{pore} profiles further supports our inhibitory model. Both tariquidar and verapamil exhibit pronounced energy minima near the cavity gate region (at $Z' \approx -1.2\ nm$ and $Z' \approx -0.6\ nm$, respectively) and neither shows an energy barrier across the transmembrane pore (17). In our inhibitory model, tariquidar might be trapped outside the cavity, preventing its removal via ABCB1-mediated efflux. This variation pattern is also observed in the PMF_{pore} profile of TPGS, suggesting that TPGS and tariquidar may have a common inhibition mechanism. In contrast, verapamil fully enters the cavity, which has a deeper binding site than that of tariquidar while shallower than those of substrates without inhibitory effects. These findings can explain why tariquidar and TPGS act as potent inhibitors with minimal or no transport activity, and also the dual roles of verapamil as both an inhibitor and substrate. Furthermore, we propose that π-π stacking between the aromatic systems of kaempferide and TPGS and the aromatic residues lining the "access tunnel" contributes significantly to the binding free energy at this amphiphilic site, as evidenced by the deep energy minima in their respective PMF_{pore} profiles derived from coarse-grained MD simulations. In contrast, such interactions are absent for andrographolide and valproic acid, which lack aromatic systems, correlating with their PMF_{pore} landscapes that exhibit elevated barriers or shallower minima rather than stabilizing troughs. This biophysical observation aligns with SHAP analyses from the MolMM model, which highlight aromatic descriptors, particularly those involving π-electron systems, as key positive contributors to inhibitor binding predictions. Cation-π interactions between cationic motifs in inhibitors and aromatic residues further enhance binding stability at the "access tunnel" (31). These cationic motifs can further enhance binding with cellular membranes (32–34), facilitating inhibitor accumulation at the lipid interface and potentially amplifying their functional impact on ABCB1. This mechanism elucidates the positive correlation between cationic features and inhibitor classification outcomes observed in SHAP analyses.

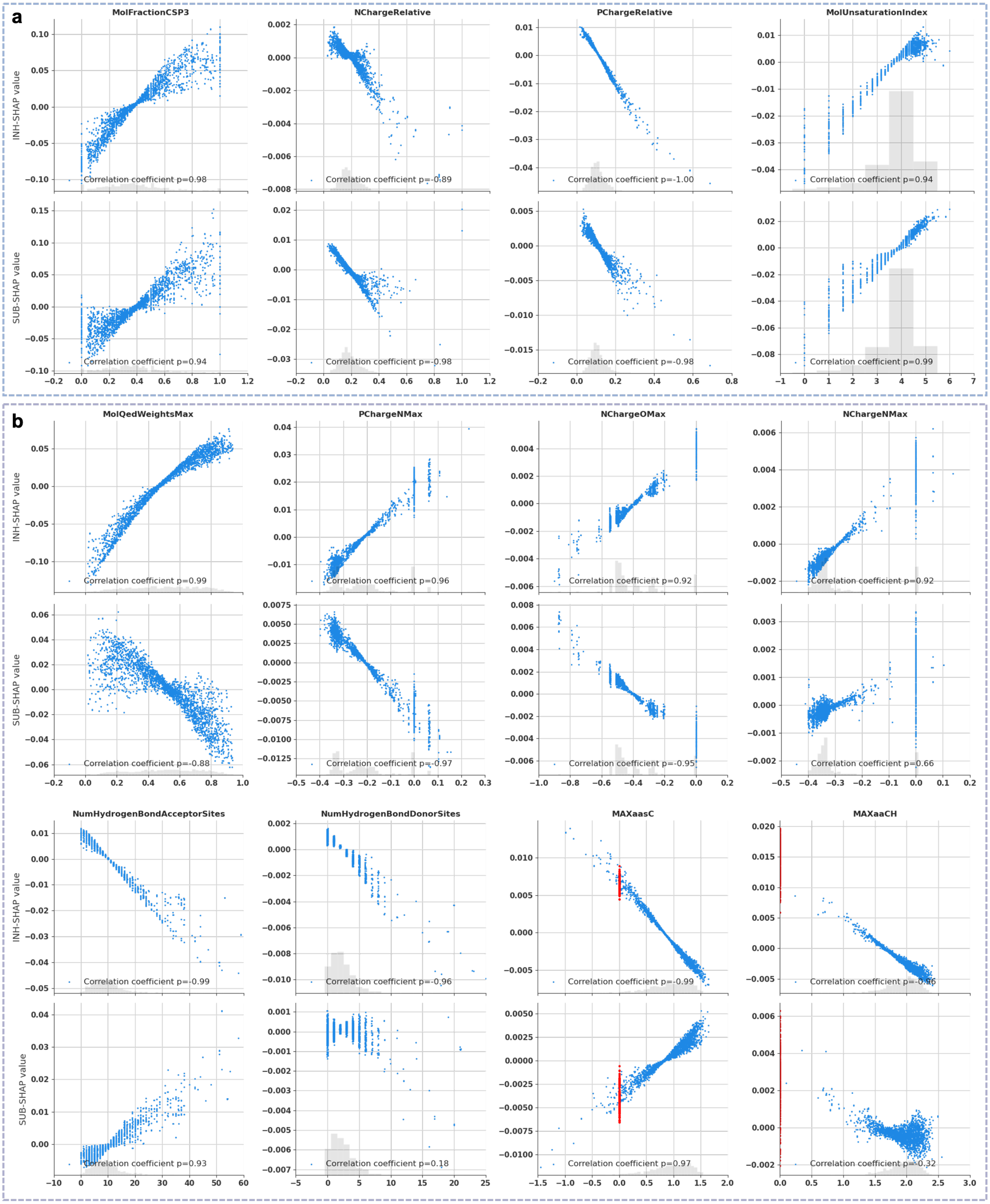

Fig. S1. SHAP dependence plots for inhibitor and substrate predictions.

Table S1. Details of our collected ABCB1 inhibition and transport assay records.

Assay format	Dataset size[2]	File path[3]	Ref.
		Unannotated records[1]	
In vitro inhibition	2,247(1,389:858)	`MolMM/csv/data_classes/data_paper/inhibitors_main*`	(3)
In vitro inhibition	1,918(1,169:749)	`MolMM/csv/data_classes/data_paper/*_multiclass*`	(35)
In vitro transport	521(451:70)		(35)
In vitro inhibition	333(259:74)	`MolMM/csv/data_classes/data_paper/*_metra*`	(36)
In vitro transport	899(489:410)		(36)
In vitro inhibition	1,748(1,588:160)	`MolMM/csv/data_classes/*_ochem`	(37)
In vitro transport	2,451(1,567:884)		(37)
		Annotated records[1]	
In vitro inhibition	1,181(283)	`MolMM/csv/data_inh_refine/inhibitors_refine*`	(38)
In vivo inhibition	200(67)	`MolMM/csv/data_inh_refine/inhibitors_vivo*`	(38)
In vitro transport	2,520(1,236)	`MolMM/csv/data_sub_refine/substrates_refine*`	(39)

[1] Annotated records include both experimental metadata and results, and unannotated records contain classification results but lack experimental metadata. Available in the GitHub repository at https://github.com/PatrickSu1101/MolMM

[2] Data is shown as "Compound number(Positive sample number: negative sample number)" in unannotated records, and as "Record number(Compound number)" in annotated records.

[3] Datasets are available in the paths of our provided codes.

Table S2. Critical screening criteria of experimental metadata.

Assay	Metadata	Screening items
		In vitro
**Inhibition**	Measured metrics	IC_{50}, K_i
	Cell lines[1]	Caco-2 cells, MDR1-containing cells, recombinant-containing cells
	Probe Substrates[2]	Calcein AM (CaAM)[3], Digoxin[3], Hoechst-33342&Colchicine (SiH)[3], Rhodamine-123 (SiR)[3], Vinblastine (SiV), Daunorubicin&Doxorubicin (SiD), Paclitaxel (SiP), Other substrates
**Efflux**	Measured metrics	ER (Efflux ratio)
	Cell lines	Caco-2 cells
**Common**	pH	7.4
		In vivo
**Inhibition**	Measured metrics[3]	$AUC_{Digoxin}$
	Substrate probes	Digoxin

[1] **X**-containing cells mean those cell lines with names containing strings **X**.
[2] ABCB1 Inhibitors are defined as those increasing $AUC_{Digoxin} \geq 1.25\ fold$ (see the January 2020 FDA guidance for industry entitled *Clinical Drug Interaction Studies — Cytochrome P450 Enzyme- and Transporter-Mediated Drug Interactions*).
[3] denotes the canonical substrate probes, that is, widely used in relevant studies.

Table S3. Substrate and inhibitor labels for the overlapping data among noisy-labeled, refined, and ground-truth datasets.

Compound	Substrate			Inhibitor		
	GTD	Noisy-labeled	SUB	GTD	Noisy-labeled	INH
Paclitaxel	True	True	True	False	True	False
Vinblastine	True	True	True	False	True	
Hoechst 33342	True	True		False	False	
Morphine	True	True		False		False
Nicardipine		False	False	True	True	True
Rhodamine 123	True	True				
Colchicine	True	True	True	False		False
Tariquidar			True	True	True	True
Elacridar		False		True	True	True
Verapamil	True	True		True	True	True
Doxorubicin	True	True				
Daunorubicin	True	True	True	False	True	False

References

1. V Poongavanam, N Haider, G Ecker, Fingerprint-based in silico models for the prediction of p-glycoprotein substrates and inhibitors. *Bioorganic Medicinal Chem.* **20**, 5388–5395 (2012).
2. F Klepsch, P Vasanthanathan, G Ecker, Ligand and structure-based classification models for prediction of p-glycoprotein inhibitors. *J. Chem. Inf. Model.* **54**, 218–229 (2014).
3. M Yang, et al., Development of in silico models for predicting p-glycoprotein inhibitors based on a two-step approach for feature selection and its application to chinese herbal medicine screening. *Mol. Pharm.* **12**, 3691–3713 (2015-10-05) Publisher: American Chemical Society.
4. Y Zhang, J Wu, Y Kang, T Hou, A multimodal contrastive learning framework for predicting p-glycoprotein substrates and inhibitors. *J. Pharm. Analysis* p. 101313 (2025-04-16).
5. Y Shirasaka, et al., Evaluation of human p-glycoprotein (MDR1/ABCB1) ATPase activity assay method by comparing with in vitro transport measurements: Michaelis-menten kinetic analysis to estimate the affinity of p-glycoprotein to drugs. *Biol. Pharm. Bull.* **29**, 2465–2471 (2006).
6. M Ansbro, S Shukla, S Ambudkar, S Yuspa, L Li, Screening compounds with a novel high-throughput ABCB1-mediated efflux assay identifies drugs with known therapeutic targets at risk for multidrug resistance interference. *PLoS ONE* **8** (2013).
7. S Lazzaro, et al., Translatability of in vitro inhibition potency to in vivo p-glycoprotein mediated drug interaction risk. *J. Pharm. Sci.* **112**, 1715–1723 (2023).
8. J Bentz, et al., Variability in p-glycoprotein inhibitory potency (IC50) using various in vitro experimental systems: Implications for universal digoxin drug-drug interaction risk assessment decision criteria. *Drug Metab. Dispos.* **41**, 1347–1366 (2013-07-01).
9. JK Zolnerciks, CL Booth-Genthe, A Gupta, J Harris, JD Unadkat, Substrate- and species-dependent inhibition of p-glycoprotein-mediated transport: Implications for predicting in vivo drug interactions. *J. Pharm. Sci.* **100**, 3055–3061 (2011-08-01).
10. WX Shen, et al., Out-of-the-box deep learning prediction of pharmaceutical properties by broadly learned knowledge-based molecular representations. *Nat. Mach. Intell.* **3**, 334–343 (2021-04) Number: 4 Publisher: Nature Publishing Group.
11. C Szegedy, et al., Going deeper with convolutions in *2015 IEEE Conference on Computer Vision and Pattern Recognition (CVPR)*, eds. C Szegedy, et al. pp. 1–9 (2015).
12. DP Kingma, J Ba, Adam: A method for stochastic optimization. *arXiv preprint arXiv:1412.6980* (2014).
13. VR Joseph, Optimal ratio for data splitting. *Stat. Analysis Data Mining: An ASA Data Sci. J.* **15**, 531–538 (2022-08-01) Publisher: John Wiley & Sons, Ltd.
14. M Mahsereci, L Balles, C Lassner, P Hennig, Early stopping without a validation set. *arXiv preprint arXiv:1703.09580* (2017).
15. A Paszke, Pytorch: An imperative style, high-performance deep learning library. *arXiv preprint arXiv:1912.01703* (2019).
16. N Subramanian, K Condic-Jurkic, AE Mark, ML O'Mara, Identification of possible binding sites for morphine and nicardipine on the multidrug transporter p-glycoprotein using umbrella sampling techniques. *J. Chem. Inf. Model.* **55**, 1202–1217 (2015-06-22) Publisher: American Chemical Society.
17. N Subramanian, A Schumann-Gillett, AE Mark, ML O'Mara, Probing the pharmacological binding sites of p-glycoprotein using umbrella sampling simulations. *J. Chem. Inf. Model.* **59**, 2287–2298 (2019-05-28) Publisher: American Chemical Society.
18. Y Qi, et al., CHARMM-GUI martini maker for coarse-grained simulations with the martini force field. *J. Chem. Theory Comput.* **11**, 4486–4494 (2015-09-08) Publisher: American Chemical Society.
19. PC Hsu, et al., CHARMM-GUI martini maker for modeling and simulation of complex bacterial membranes with lipopolysaccharides. *J. Comput. Chem.* **38**, 2354–2363 (2017-10-15) Publisher: John Wiley & Sons, Ltd.
20. MA Lomize, AL Lomize, ID Pogozheva, HI Mosberg, OPM: Orientations of proteins in membranes database. *Bioinformatics* **22**, 623–625 (2006-03-01).
21. PCT Souza, et al., Martini 3: a general purpose force field for coarse-grained molecular dynamics. *Nat. Methods* **18**, 382–388 (2021-04-01).
22. P Eastman, et al., OpenMM 7: Rapid development of high performance algorithms for molecular dynamics. *PLOS Comput. Biol.* **13**, e1005659 (2017-07-26) Publisher: Public Library of Science.
23. MJ Abraham, et al., GROMACS: High performance molecular simulations through multi-level parallelism from laptops to supercomputers. *SoftwareX* **1-2**, 19–25 (2015-09-01).
24. L Kagami, A Wilter, A Diaz, W Vranken, The ACPYPE web server for small-molecule MD topology generation. *Bioinformatics* **39**, btad350 (2023-06-01).
25. J Wang, RM Wolf, JW Caldwell, PA Kollman, DA Case, Development and testing of a general amber force field. *J. Comput. Chem.* **25**, 1157–1174 (2004-07-15) Publisher: John Wiley & Sons, Ltd.
26. A Jakalian, DB Jack, CI Bayly, Fast, efficient generation of high-quality atomic charges. AM1-BCC model: II. parameterization and validation. *J. Comput. Chem.* **23**, 1623–1641 (2002-12-01) Publisher: John Wiley & Sons, Ltd.
27. R Alessandri, et al., Martini 3 coarse-grained force field: Small molecules. *Adv. Theory Simulations* **5**, 2100391 (2022-01-01) Publisher: John Wiley & Sons, Ltd.
28. A Alam, J Kowal, E Broude, I Roninson, K Locher, Structural insight into substrate and inhibitor discrimination by

human p-glycoprotein. *Science* **363**, 753–756 (2019).
29. K Nosol, et al., Cryo-EM structures reveal distinct mechanisms of inhibition of the human multidrug transporter ABCB1. *Proc. Natl. Acad. Sci. United States Am.* **117**, 26245–26253 (2020).
30. C Martin, et al., Communication between multiple drug binding sites on p-glycoprotein. *Mol. Pharmacol.* **58**, 624–632 (2000-09-01) Publisher: Elsevier.
31. J Gallivan, D Dougherty, Cation-π interactions in structural biology. *Proc. Natl. Acad. Sci. United States Am.* **96**, 9459–9464 (1999).
32. Y Su, S Li, M Hong, Cationic membrane peptides: Atomic-level insight of structure-activity relationships from solid-state NMR. *Amino Acids* **44**, 821–833 (2013).
33. C Anderson, A Cardenas, R Elber, L Webb, Preferential equilibrium partitioning of positively charged tryptophan into phosphatidylcholine bilayer membranes. *J. Phys. Chem. B* **123**, 170–179 (2019).
34. S Jakka, G Mugesh, Emerging role of noncovalent interactions and disulfide bond formation in the cellular uptake of small molecules and proteins. *Chem. - An Asian J.* **20** (2025).
35. L Mora Lagares, N Minovski, M Novič, Multiclass classifier for p-glycoprotein substrates, inhibitors, and non-active compounds. *Molecules* **24** (2019).
36. L Mak, et al., Metrabase: a cheminformatics and bioinformatics database for small molecule transporter data analysis and (q)SAR modeling. *J. Cheminformatics* **7**, 31 (2015-06-23).
37. I Sushko, et al., Online chemical modeling environment (OCHEM): web platform for data storage, model development and publishing of chemical information. *J. Cheminformatics* **3**, P20 (2011-04-19).
38. J Peng, J Yi, G Yang, Z Huang, D Cao, ISTransbase: an online database for inhibitor and substrate of drug transporters. *Database* **2024**, baae053 (2024-02-12).
39. A Gaulton, et al., ChEMBL: a large-scale bioactivity database for drug discovery. *Nucleic Acids Res.* **40**, D1100–D1107 (2012-01-01).
